# Obituary: Professor Dieter C Gruenert

**DOI:** 10.1038/cddiscovery.2016.73

**Published:** 2016-10-24

**Authors:** Giuseppe Novelli, Federica C Sangiuolo

**Affiliations:** 1 Tor Vergata University of Rome, Rome, Italy


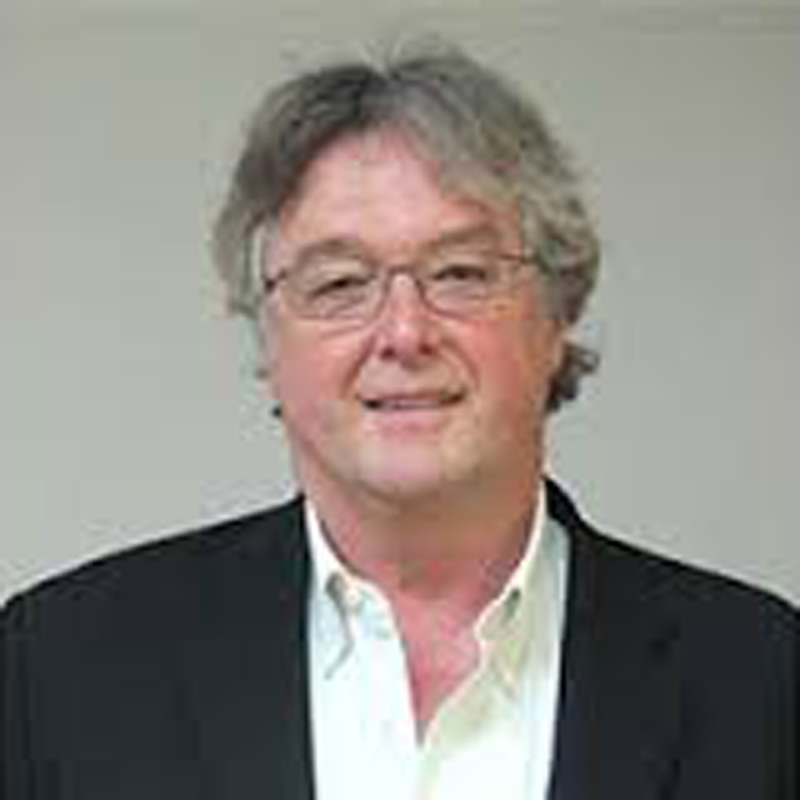


Dieter C Gruenert, who died aged 67, was a Geneticist in charge to the Department of Otolaryngology-Head and Neck Surgery of the University of California San Francisco (UCSF). He was also a member of the Eli Edythe Broad Center for Regenerative Medicine and Stem Cell Research, the Institute for Human Genetics, the Helen Diller Family Comprehensive Cancer Center, and the Cardiovascular Research Institute at UCSF.

Born in Dortmund, Germany, he received his PhD in Biophysics from UC Berkeley in 1982 and until 1984 was postdoctoral fellow in the Department of Carcinogenesis at the Swiss Institute for Experimental Cancer Research in Lausanne, Switzerland. He originally joined the faculty at UCSF in 1986, where he developed many of the human cystic fibrosis (CF) and non-CF airway epithelial cell lines used in airway disease research throughout the world. He was also the Co-Director of the Gene Therapy Core Center at UCSF from 1992 to 1999.

In 2000, he left UCSF to take the position of Professor of Medicine and Director of the Division of Human Molecular Genetics at the University of Vermont, and then returned to San Francisco in 2003 to take position as Senior Scientist at the California Pacific Medical Center Research Institute.

Dieter Gruenert can be considered the pioneer of gene editing; he developed a prototype of targeted genome editing called Small Fragment Homologous Replacement (SFHR). SFHR uses small DNA fragments (SDFs) to obtain homologous replacement in recipient cells. After entering the cells, it exchanges the sequences of these SDFs with the endogenous (genomic or episomal), through homologous recombination (HR) and DNA repair. SDFs recognizing and annealing itself to the mutated target, promotes the formation of a D-loop structure. This hybrid structure activates the endogenous protein complex involved in DNA repair by HR and allows the SDF to be integrated within the genomic DNA.

Together with his group, we were able to target gene alterations in mammalian cells *in vitro* and *in vivo*, suggesting a broad range of utility in terms of target genes and cell types able to support SFHR. Unfortunately, SFHR was limited by a low and variable frequency of correction, ranging from 1% to 5% *in vitro* and about 0.1% *in vivo*. However, this technique paved the way to the extraordinary and more efficient CRISPR/Cas9 method.

During the last years, Dieter’s group focused attention on gene editing approaches using induced pluripotent stem cells (iPSCs) in order to develop novel therapeutic strategies for inherited diseases.

Dr Gruenert published more than 150 publications, holds 5 patents, and has given 240 invited presentations worldwide; he reviews grants for numerous national and international agencies and he is also on the Editorial Boards of a number of prestigious scientific journals, such as Member of Oligonucleotide Therapies Society (board director since 2008) and American Association Gene Therapy (chair 2006–2008).

He has passed short, intense periods as visiting professor at the Necker Hospital in Paris (France) and in Tor Vergata University in Rome. Moreover, his current appointment to the Commission for the National Agency for Evaluation of Professor and Associate Professor Candidates for the Italian Ministry for Education, Universities and Research in Italy is a reflection of the impact his work has had internationally.

He mainly worked:

1. On gene targeting, and on understanding the mechanistic underpinnings of homologous replacement in human somatic cells;
2. On non-viral DNA delivery systems (liposomes, polyamidoamines, polyethyleneimines, microinjection, and electroporation for *in vitro*, *in vivo*, and *ex vivo* DNA delivery);
3. On cancer gene therapy, by studying connexin genes in both transformed and non-transformed cells to characterize the role of intercellular communication in modulating neoplastic progression;
4. On stem cell-based tissue repair, evaluating in embryonic and induced stem cells (derived mainly from cystic fibrosis and sickle cell disease patients) the determinants for multilineage differentiation.

His last paper, published on January 2016 *on Molecular Therapy—Nucleic Acids*, reports an interesting finding on the correction of the most frequent CF mutation, the F508del, in airway epithelial cells derived from CF-iPSCs. The strategy was really innovative, based on small/short DNA fragments (SDFs) and sequence-specific TALENs: when differentiated into endoderm/airway-like epithelial cells, a wild-type cAMP-dependent Cl ion transport was recovered.

Dieter’s contribution to genetics and biology was not confined only to his deep research work. He was a generous educator who helped tens of PhD students to graduate. He was a very dedicated teacher whose lectures were very clear and well appreciated by his students.

Dieter was also a human being with a rich culture and generous spirit. He was also a good friend. He always held discussions with friends and students on extraordinary and amazing subjects, and also discussed with passion and deep knowledge on a large number of favorite science subjects.

His wife and three sons survive him. He was always very proud of his passion towards science and excellence.

He will be sorely missed by generations of students, by his many peers, friends, and collaborators in the scientific community.

